# Recommendations for return to sports after total hip arthroplasty are becoming less restrictive as implants improve

**DOI:** 10.1007/s00402-020-03691-1

**Published:** 2020-12-01

**Authors:** T. Vu-Han, S. Hardt, R. Ascherl, C. Gwinner, C. Perka

**Affiliations:** 1 Department of Orthopaedic Surgery and Traumatology, Charité Berlin University Hospital, Chariteplatz 1, 10117 Berlin, Germany; 2 Department of Orthopaedic Surgery and Arthroplasty Nordoberpfalz AG, Krankenhaus Tirschenreuth, St.-Peter-Str. 31, 95643 Tirschenreuth, Germany

**Keywords:** Return to sports, Total hip arthroplasty, Osteoarthritis, Joint replacement

## Abstract

**Introduction:**

Total hip arthroplasty (THA) surgeries are expected to exponentially increase in the upcoming years, likely because of the overall broader indication of THAs. With these developments, an increasing number of younger (< 50 years) and active patients will receive surgical interventions, and expectations for an active lifestyle will accordingly increase. In addition, surgeons now have a growing array of techniques and implant materials to choose from. Despite these developments, evidence to provide the best standard-of-care to patients with high expectations for return to sports (RTS) is scarce and urgently needed. What recommendations do arthroplasty surgeons currently make to patients with high return to sports expectations, what factors may influence their recommendations and what surgical techniques and implant specifications are considered favorable in the treatment of patients with a more active lifestyle? This study was conducted to analyze the current recommendations, patient assessment, and patient counseling after THA to identify trends and relevant factors for surgical decision-making in patients with high-RTS expectations.

**Material and methods:**

We designed a questionnaire comprising five general items and 19 specific items that included 46 sub-items for hip arthroplasty and conducted a survey among 300 German surgeons specialized in arthroplasty at the German Arthroplasty Society (AE) to assess expert opinions, recommendations, surgical decision-making, and patient counseling for patients with high expectations for RTS after THA.

**Results:**

The majority of surgeons (81.9%) were in favor of RTS after THA. Risks associated with sports after THA were considered minimal (1%), with periprosthetic fractures ranking highest, followed by hip dislocation and polyethylene wear. Some surgical decision-making was influenced by high-RTS expectations in regard to implant fixation, stem type, femoral head diameter, and bearing-surface tribology. We observed an increasingly liberal counseling of patients for high-impact sports.

**Conclusion:**

With the improvement of implants and surgical techniques, surgeons are more willing to encourage patients to adopt a more active lifestyle. However, the true long-term limitations need further investigation in future studies.

**Level of evidence:**

5 Expert opinions.

**Electronic supplementary material:**

The online version of this article (10.1007/s00402-020-03691-1) contains supplementary material, which is available to authorized users.

## Introduction

Hip arthroplasties are on the rise. Current projections suggest an expected increase of up to 284% for total hip arthroplasty (THA) within the next 20 years [22, 29, 38, 58]. Improved surgical techniques and higher quality of implants have resulted in more cost-effective medical interventions and broader medical indication for hip replacement candidates. Furthermore, improved quality of life in the elderly as well as rising numbers of younger patients (age < 50 years) receiving THA have introduced heightened expectations with respect to physical activity and participation in sports [15, 46, 52, 53]. While surgeons and physicians are typically expected to provide recommendations and counseling to patients regarding an active lifestyle [9, 63], data on complications associated with physical activity provide little evidence regarding long-term outcomes [43]. In addition, surgeons have a growing array of different implants and surgical techniques to weigh and choose from, and it is unknown whether patients with high return to sports (RTS) expectations should receive specific hip arthroplasty treatment in anticipation of higher load- and weight-bearing of the implants. Furthermore, patient surveys suggest that patients’ reluctance to RTS is mostly due to anxiety rather than pain [2]. Recommendations and guidelines in surgical decision-making and patient counseling to provide the current best standard-of-care to patients are therefore urgently needed. With approximately 450,000 primary THAs performed in Germany in 2018 [21] and 309 THAs per 100,000 population, Germany is one of the leading countries in hip arthroplasty, and ranked ahead of Switzerland and Austria in 2019 [1]. We asked what recommendations arthroplasty surgeons make to patients with high return to sports expectations, what factors may influence their recommendations and what surgical techniques and implant specifications are considered favorable in the treatment of patients with a more active lifestyle. The objective of this study was to capture and evaluate the current recommendations, patient assessment, and patient counseling among surgeons in Germany, who specialized in arthroplasty of the hip and knee. Furthermore, we aimed to identify current recommendations and relevant factors for surgical decision-making in patients with high-RTS expectations and compared those with evidence in current literature.

## Materials and methods

We performed a survey among surgeons specialized in hip and knee arthroplasty who were members of the German Arthroplasty Society (AE, Arbeitsgemeinschaft für Endoprothetik)—Germany’s largest and leading society of hip and knee arthroplasty surgeons, and who attended the annual AE Meeting in December 2019. Membership approval in the AE society requires a completed residency in orthopedic and trauma surgery with sub-specialization in arthroplasty and an endorsement by an AE member. AE members must perform at least 50 arthroplasty surgeries per annum to maintain status. We designed an extensive questionnaire that consisted of five general items and 19 specific items that included 46 sub-items for hip arthroplasty. The questionnaire items were conceptualized in cooperation with the presidium of the German Hip and Knee Arthroplasty Society (AE) in multiple iterations to ensure relevance of the questions in the field. The questionnaire was designed to assess pre-operative patient factors that may influence implant longevity and surgical decision-making, such as surgical planning, surgical approach, implant positioning, and fixation in patients with high-RTS expectations. Furthermore, the questionnaire assessed expert recommendations and patient counseling for RTS after hip arthroplasty. The original questionnaire, as well as an English translation, are provided in the supplemental section. In all, 300 questionnaires were distributed among hip and knee arthroplasty experts. Of these, 99 questionnaires were returned over a period of 2 months, equaling an effective response rate of 33%. In the current literature, there is no uniform consensus with respect to which type of sports are considered low and high impact. For the purpose of our questionnaire, we characterized low-impact sports as those involving smooth and gentle movements. In contrast, high-impact sports were characterized by rapid and abrupt movements with heightened risk of injury, especially without training. The returned questionnaires were analyzed using R Version 3.6.3 by The R Foundation for Statistical Computing and figures were produced using the package ggplot2 [62]. Each returned questionnaire received a unique identification number (ID) and answers were coded into an R data frame (see supplementary material). Missing values were coded as ‘NA’. For multiple choice questions, the absolute counts were given. This study did not require ethical approval, as no human subjects were involved, and participation in the survey was voluntary.

## Results

Our primary research question was to identify current recommendations made by arthroplasty surgeons to patients with high-RTS expectations. The secondary research goal was to determine factors that may influence RTS capacity and whether surgeons prefer certain surgical techniques and THA implant specifications to treat patients with an active lifestyle.

### Survey participants

Overall, 82.8% survey participants had more than 10 years’ surgical experience, and 52.5% more than 20 years’ surgical experience.

### Perioperative patient assessment for THA

**Fig. 1:**
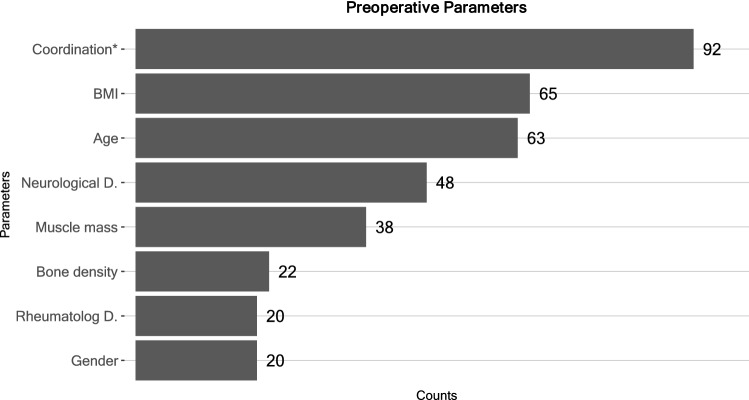
Pre-operative parameters and their influence on return to sports (RTS) after THA. *Coordination = i.e., muscle coordination, experience with the type of sport. *D* disease or precondition. Multiple choices were possible; absolute counts are labeled

Overall, 77.8% surgeons included assessment of the patient’s physical activity level in their standard pre-operative patient work-up. The most frequently selected factors that influenced post-operative RTS capacity included coordination (i.e., previous experience in a specific type of sport), body mass index (BMI), and age, and less often, neurological preconditions and muscle mass (Fig. 1).

### Risk assessment in patients with high expectations for RTS after THA

**Fig. 2:**
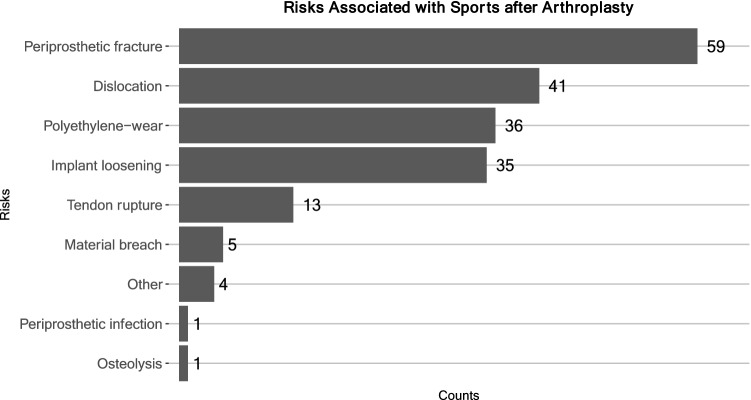
Estimated risks associated with sports after THA. A maximum of two selections were possible; absolute counts are labeled

**Fig. 3:**
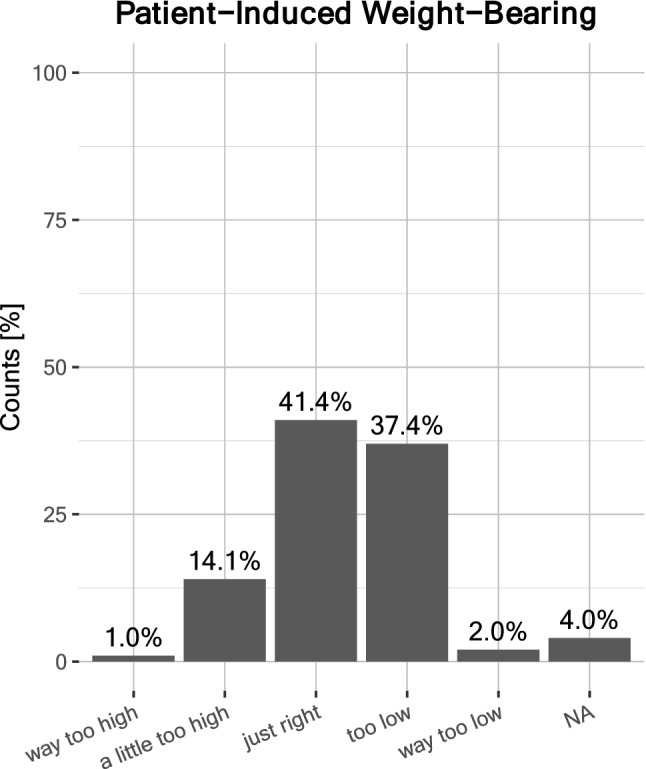
Estimated patient-induced weight-bearing on the hip implant

**Fig. 4:**
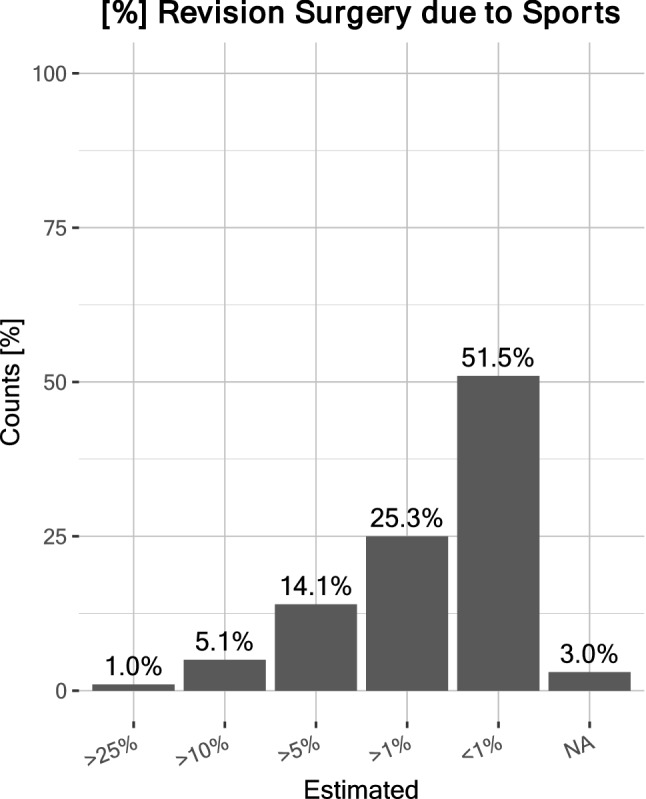
Estimated prevalence of revision surgery due to sports after THA. The majority of surgeons considered revision surgery due to sports to be minimal: < 1% or ~ 1%

According to most surgeons, RTS after hip arthroplasty posed the greatest risk for periprosthetic fractures and dislocation, while polyethylene wear and implant loosening ranked third and fourth, respectively (Fig. 2). Briefly, 55.6% surgeons considered physical activity as important and 26.3% considered it very important, amounting to 81.9% in favor of physical activity after arthroplasty. However, 13.1% considered it unimportant and 5.1% stated that it did not matter. Accordingly, 58.6% surgeons did not think that RTS had a negative impact on the longevity of the hip implant; 26.3% did; 12.1% were ‘undecided’; 2% felt that it ‘did not matter’, and 1% responded ‘NA’. Conversely, 79.8% expected a positive impact of sports on the longevity of hip implant, and 15.2% did not. Moreover, 2% surgeons considered their patients’ weight bearing on the implant was ‘way too low’, 37.4% said ‘too low’, 41.4% considered it ‘just right’, 14.1% said weight bearing was ‘a little too high’, and 1% felt it was ‘way too high’ (Fig. 3). The data suggested that many experts (~ 39.4%) would encourage patients to increase physical activity overall. Most surgeons (51.5% and 25.3%, respectively) estimated that the prevalence of sports-related revision surgery after hip arthroplasty caused by stress overload on the implant was ‘< 1%’ and ‘> 1%’, respectively (Fig. 4).

### Surgical decision-making in patients with high RTS after THA

**Fig. 5:**
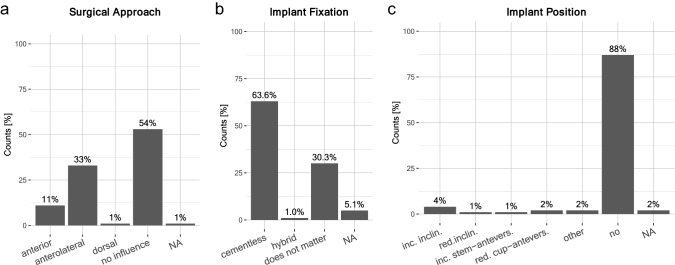
Surgical decisions in patients with high-RTS expectations: **a** Preferred surgical approach—‘No influence’ on the surgical approach (54%) or ‘anterolateral’ (33%) or ‘anterior approach’ (11%). **b** Preferred implant fixation—‘cementless’ (63.6%) or ‘does not matter’ (30.3%). **c** Preferred implant position—‘no influence’ (88%), other options included increased inclination (4%), reduced inclination (1%), increased stem-anteversion (1%), and reduced cup-anteversion (2%)

**Fig. 6:**
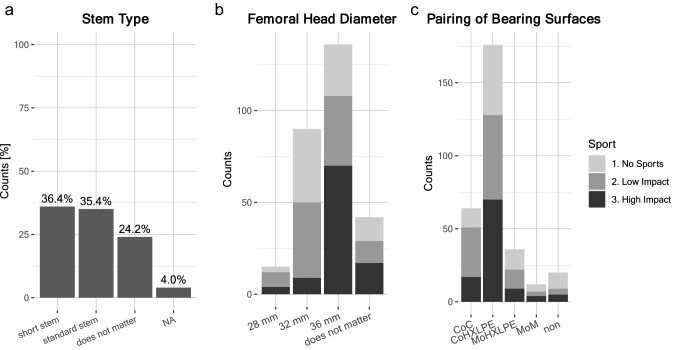
Implant specific decisions in patients with high-RTS expectations: **a** Preferred stem type—‘short stem’ (36.4%) or ‘standard stem’ (35.4%) (also known as straight stem) or ‘does not matter’ (24.2%). **b** Preferred femoral head diameter (multiple choices possible, numbers given as absolute counts): 28 mm (‘High-impact’: 4 counts, ‘Low-impact’: 8 counts, ‘No Sports’: 3 counts). 32 mm (‘High-impact: 9 counts, ‘Low-impact’: 41 counts, ‘No Sports’: 40 counts). 36 mm (‘High-impact’: 70 counts, ‘Low-impact’: 38 counts, ‘No Sports’: 28 counts). **c** Preferred pairing of bearing surfaces (multiple choices possible, numbers given as absolute counts)—CoC (‘High-impact’: 17 counts, ‘Low-Impact’: 34 counts, ‘No Sports’: 13 counts). CoHXLPE (‘High-impact’: 70 counts, ‘Low-Impact’: 58 counts, ‘No Sports’: 48 counts). MoHXLPE (‘High-impact’: 9 counts, ‘Low-Impact’: 13 counts, ‘No Sports’: 14 counts). MoM (‘High-impact’: 4 counts, ‘Low-Impact’: 3 counts, ‘No Sports’: 5 counts)

Next, the aim of this study was to assess whether high patient expectations for RTS after hip arthroplasty influenced surgical decision-making. Surgical approach: For the majority of surgeons (53.5%), high expectations of patients for RTS after arthroplasty did not influence their choice of surgical approach: 33.3% preferred an ‘anterolateral’ approach (also called OCM or modified Watson–Jones approach) and 11.1% preferred an ‘anterior’ surgical approach (Fig. 5a). Implant fixation: For patients with high expectations for RTS the majority of surgeons, 63.6%, preferred ‘cementless’ implant fixation. 30.3% of surgeons stated it played no role, 5.1% were ‘undecided’ (Fig. 5b). Implant positioning: The positioning of the implant was not influenced by patient expectations for RTS, as stated by 87.9% of surgeons. 4.0% preferred increased inclination and 2.0% reduced cup-anteversion (Fig. 5c). Stem type: Regarding stem type, 36.4% preferred ‘short-stems’ and 35.4% preferred ‘standard stems’ in patients with high expectations for RTS. At least 24.2% stated that the choice of stem type was not influenced by RTS expectations (Fig. 6a). Femoral head diameter: Most surgeons showed a preference for femoral head diameters of ‘36 mm’ (Fig. 6b) in patients with high expectations for RTS in case of high-impact sports, and at least ‘32 mm’ for return to low-impact sports. Bearing surface tribology: Ceramic-on-highly-cross-linked-polyethylene (CoHXLPE) was the preferred pairing for bearing surfaces in high-impact sports and also ranked highly for low-impact, as well as no sports; ceramic-on-ceramic (CoC) ranked second (Fig. 6c).

### Recommendations for RTS after THA

**Fig. 7:**
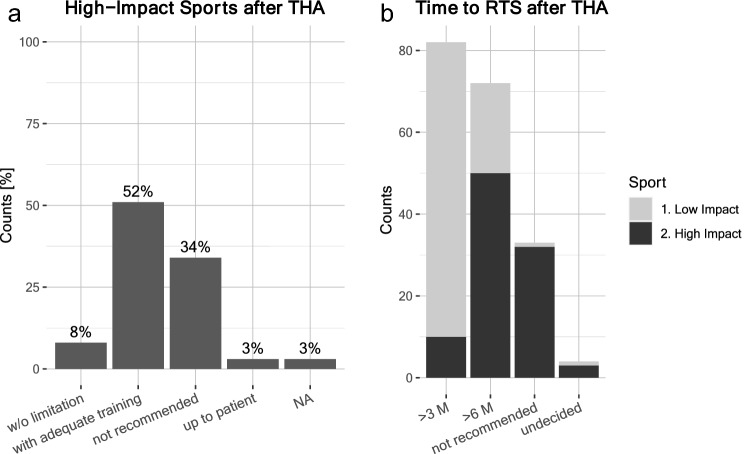
**a** Recommendations for high-impact sports after THA, ‘without limitation’ 8%, ‘with adequate training’ 52%, ‘not recommended’ 34%, ‘up to the patient’ 3%, NA 3%. **b** Recommended time to return to sports after THA for low- vs. high-impact sports (numbers as absolute counts): High-Impact Sports—‘ > 6 M’: 50 counts, ‘ > 3 M’: 10 counts, ‘not recommended’: 32 counts, ‘undecided’: 3 counts vs. Low-Impact Sports—‘ > 6 M’: 22 counts, ‘ > 3 M’: 72 counts, ‘not recommended’: 1 count, ‘undecided’: 1 count.

The results of the survey showed uniform recommendations for return to low-impact sports, which was recommended without limitations and within 3 months after THA. In contrast, surgeons’ recommendations varied much more regarding a return to high-impact sports after THA. 51.5% surgeons advised a return to high-impact sports if the patient received adequate training, 8.1% even without limitations (Fig. 7a). Still, 34.3% surgeons did not recommend high-impact sports at all (3% considered it up to the patient). In case of high-impact sports, most experts recommended at least 6 months before RTS (Fig. 7b).

**Fig. 8:**
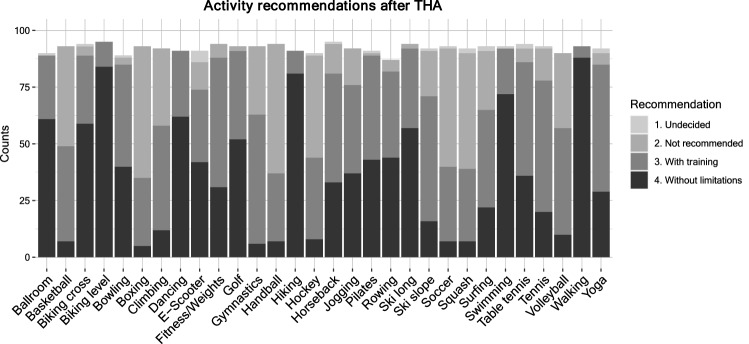
Specific activity recommendations after THA, *y*-axis: numbers as absolute counts, *x*-axis: type of activity/sport (Ballroom was abbreviated for ballroom dancing). Recommendations ‘without limitations’ (dark grey), ‘with adequate training’ (medium grey), ‘not recommended’ (light grey), ‘undecided’ (lightest grey)

The results of this survey show that sports including basketball, boxing, soccer, gymnastics, handball, hockey, squash, climbing, volleyball, tennis, and skiing on slopes were mostly not recommended or only with adequate training (Fig. 8). In contrast, walking, swimming, hiking, and level biking were among the activities that the vast majority of surgeons recommended without limitations or training. Recommendations seemed to vary more for sports such as ballroom dancing, cross country biking, bowling, dancing, e-scooters, fitness/weights, golf, horseback riding, jogging, Pilates, cross country skiing, table tennis, and yoga where the sport was either recommended without limitations or with adequate training.

## Discussion

Our study results showed that preoperative physical activity of the patient was part of a standard patient interview for the majority of surgeons. Previous studies suggest that the level of preoperative physical activity of the patient correlates with patient expectations for post-operative RTS and return to physical activity (RPA) [26, 32, 35, 37]. Along with current trends toward younger and more active patient clientele receiving THA [43], managing and counseling patient expectations after THA may become an important task for surgeons and physicians, who will need to provide answers despite the lack of evidence in the literature [9].

Among the risks associated with RTS after hip replacement, wear-related implant failure only ranked third after periprosthetic fractures and hip dislocation among expert opinions. The shift away from the previous assumption that high-activity levels seen in younger, active patients would impact long-term wear-related implant survivorship [35] may reflect the improvement in implants. Early implants were accompanied by restrictive recommendations with regard to physical activity [10]. Polyethylene wear and implant abrasion are used to pose a viable risk for patients with hip or knee replacement. The risk of abrasion and implant loosening was especially true for the metal-on-metal (MoM) pairings [5, 20], which were associated with adverse local tissue reactions (ALTR) and aseptic lymphocytic vasculitis-associated lesions (ALVAL) [14]. More recent publications as well as our results suggest a growing trend toward liberal counseling of patients with regard to RTS after THA [7, 40, 44, 60]. Recent publications support that lower wear rates were observed in the pairing of CoHXLPE [3]. CoC bearings have shown lower wear rates than polyethylene, and the improvement of ceramic materials has lowered the risks of ceramic fractures; however, long-term data are not yet available. Notably, incidences of ‘squeaking’ observed in CoC bearings seem to persist, ranging between 0.5 and 20% [8, 49], and may be worth discussing with the patient.

In our survey, hip–joint dislocation ranked second among risks associated with sports after THA; however, evidence provided by the current literature is scarce. Hence, this assessment may be hypothetical at this time [48]. The trend toward larger femoral heads of 32–36 mm may further reduce the risks for hip–joint dislocation. Tsikandylakis et al. noted no benefit in hip range of movement of function in larger heads (> 36 mm), with the advantage of lower risks of revision due to dislocation in larger femoral heads [61]. Surgeons generally have to strike a balance, as larger head diameters are associated with higher volumetric and total wear rates [39]. Further improvements in implant materials may reduce these issues in the future.

Finally, our survey results showed that periprosthetic fractures ranked highest among risks associated with RTS after THA. The evidence regarding the incidence is scarce, as only two cases were reported in association with winter sports [42]; however, the sequelae in case of injury can be severe after arthroplasty [37]. Studies have shown that bone mineral density can be reduced after THA [28]. On the other hand, physical activity is known to increase overall bone density and prevent osteoporosis [16]. Given the lack of evidence for sport-associated periprosthetic fractures after THA, the high rank assigned to this risk seems relatively hypothetical at this time.

Surgeons have a growing array of different implants, materials, and surgical techniques to consider and choose from. At this time, there are no evidence-based consensus guidelines and uniform standards, and needless to say, some of the current decisions may be based on the surgeon’s personal training and preference alone. Surgical approach: In our survey, most surgeons stated that high-RTS expectations did not influence their surgical approach. Indeed, several studies have shown that surgical approach has little-to-no significant impact on RTS [34, 51]. Minimally invasive anterolateral or anterior surgical approaches reduce the detachment of soft tissue and muscles, and are hypothetically associated with reduced post-operative pain, potentially allowing an early recovery and return to physical activity. However, long-term observations in outcomes suggest comparable results to date [12, 45, 47, 65]. Overall, this is in line with the majority of surgeons’ response that high-RTS expectations from patients did not influence their choice of surgical approach. Surgeons who participated in this survey did not state whether they deviated from their standard approach in patients with high-RTS expectations. Thus, it remains unknown whether preferences for ‘anterolateral’ (33%) and ‘anterior’ (11%) approaches were a standard personal preference of surgeons. Cementless fixation: The majority of surgeons who participated in our survey showed a preference for cementless fixation, a persisting trend observed in Germany [21]. This may correlate with the notion that young and active patients have a higher probability of revision THA in their lifetime. However, cementless fixation trends are not observable in all countries; among UK surgeons, cemented stems remain a popular choice according to registry data [7]. Implant positioning: The results of our survey suggested that implant positioning was not influenced by high expectations for RTS; however, a small portion of surgeons chose increased inclination and reduced cup-anteversion. These numbers are insignificant, but may be reflective of an emerging discussion regarding the role of spinopelvic alignment in hip arthroplasty [41, 55, 59, 64]. Stem preference: Both short and standard stems ranked almost equally among surgeon preferences in our survey. These choices were in line with published clinical studies reporting no significant difference between short vs. standard stems [17, 25, 57]. Femoral head diameter: Large femoral head diameters of at least 32 mm were clearly preferred. Indeed, a trend towards large femoral head sizes has been observed over the years. The potential advantages of larger femoral head diameters are improved stability owing to increased jump distance and impingement free range of motion owing to increased head–neck ratio [14, 31]. Recent meta-analyses suggested that previously observed volumetric wear and frictional torque associated with large femoral heads may be minimal with improved materials in CoHXLPE and CoC pairing [14, 61]. Bearing surface tribology: The improved tribology of hip implants has provided a vast set of combinatorial options to the operating surgeon, wherein the most commonly used pairings include CoC, CoHXLPE, metal-on-highly-cross-linked-polyethylene (MoHXLPE), and MoM. Results from several studies investigating the advantages of each pairing, as well as the results of our survey, suggest a growing preference for CoHXLPE in patients with high-RTS expectations [27]. Our survey responses were in line with these previously reported trends.

### Return to sports after THA

Previous studies have suggested that the level of preoperative physical activity of the patient correlates with patient expectations for post-operative RTS and return to physical activity (RPA) [35]. Currently, there is no consensus regarding which type of sports are rightfully categorized as low, intermediate, or high impact with respect to bearing forces acting on the hip implant [60]. Jogging, volleyball, squash, soccer, hockey, handball, basketball, and gymnastics are often ranked among high-impact sports by previous studies and have been associated with ‘no recommendations’ after THA in earlier studies [23, 24, 36, 40, 42, 60]. Interestingly, we observed more liberal recommendations regarding high-impact sports: 52% recommended high-impact sports with adequate training, 8% recommended high-impact sports without limitations (Fig. 7a). Especially with regard to ‘jogging’, surgeons who participated in this survey recommended jogging with adequate training, some even without limitations, in contrast to earlier studies [23, 24, 36, 40, 42, 60]. This observation is in line with recent reports on this topic [7, 44], overall suggesting a trend toward a greater tolerance for high-impact sports. Only limited in vitro data exist about the biomechanical ‘upper limit’ of the possible load on implants, especially with regard to the various sports [6]. In vivo load bearing measurements have the potential to shed light on the forces at play during sports after THA; however, the true limits of implants over time remain obscure. Bergmann et al. [4] showed that repetitive unloading and simultaneous movement improve joint lubrication, while sustained high loads increase friction. These observations could fall in line with more liberal counseling for activities such as jogging and running, where repetitive unloading is the case.

The results of our survey also showed that coordination (i.e., previous experience in the type of sport) played a pivotal role during patient assessment for RTS (Fig. 1). Previous studies have observed an increase in the number of sports performed and the associated increase in stress on the endoprosthesis [13, 26, 57]. However, the translation of in vitro data to clinical recommendations continues to be extremely difficult and further studies will be required in the future. Needless to say, patients with high expectations for RTS require a disciplined and balanced physical therapy after THA, and should be counseled on available new technologies and novel applications [50].

### Limitations

This study is based on the assessment of expert opinions, i.e., surgeons specialized in arthroplasty in Germany. Expert opinions comprise empirical data as years of surgeons’ experience, surgical education, and acquired knowledge of the latest literature as well as biomechanical understanding. As such, it does not provide in vivo tested evidence. Germany is one of the leading countries in hip and knee arthroplasty [1]. This study was conducted among surgeons specialized in hip and knee arthroplasty in Germany. There may be international differences among surgeons and their training regarding surgical approach and fixation methods. Surgeons’ decisions are often based on their experience and training [56]. To better understand the surgeons’ recommendations, we compared the answers to our survey with the current literature to provide further evidence that may have led to the trends and preferences observed in our survey results.

Although sports after THA are associated with higher wear rates, no study to date has linked sports with higher rates of THA revisions, to the best of our knowledge [30, 33, 54]. A recent review by Meek et al. [43] observed that recent evidence suggests that the “disregard of precautions may be beneficial”. The discrepancy between the observations is not yet fully understood. Previous studies investigating the RTS rate after THA suggest that patients tend to resume low-impact sports after THA; high-impact sports after THA are still rare [11, 18, 30]. The survey results suggest that most patients are reluctant to increase physical activity because of the fear of stressing the implant or having been counseled by their doctor, surgeon, or therapist [33]. Given this reality, it does not seem surprising that many surgeons in our survey considered the patient-induced weight-bearing stress on hip implants rather low. Most patients could probably be encouraged to engage in higher levels of physical activity with regard to the capacities of their hip implant as well as secondary health benefits [19, 32]. With improved implant materials, surgeons’ recommendations regarding RTS and physical activity after THA have become more liberal. Whether these recommendations are justified remains to be seen, as long-term outcomes are not available. Nevertheless, it is important to note the discrepancy between expert recommendations and patient reality, as there are only limited techniques available to test the in vivo weight-bearing forces acting on the implants over time. The data acquired in national implant registries will likely provide the data needed to refine our recommendations in the future.

It is important to note that the field of arthroplasty is highly dynamic; patient clientele, surgical techniques, and available implants are typically rapidly evolving. The outcomes related to these changes, measured by implant longevity and long-term patient satisfaction, will only become evident with a delay of 20–30 years.

## Conclusion

Arthroplasty surgeons’ recommendations for RTS after hip arthroplasty are becoming more liberal with implant improvements. At the same time, there is a lack of evidence regarding the true limitations of these implants, given that in vivo studies of biomechanical load bearing over the course of 20–30 years are not available yet. In turn, the surgeons participating in our survey as well as the current literature have reported only minimal sports-associated complications after THA, thereby further encouraging the impression that improved implants are safe. At the same time, surgeons and patient-reported surveys suggest that patient’s weight-bearing on the implants is rather low. These factors could likely encourage misinformation regarding the true limitations of the implants. Despite an extensive array of available implants, materials, and surgical techniques, guidelines and recommendations regarding pre-operative patient assessment, surgical decision-making, and counseling of patients with high-RTS expectations do not exist. Our observations in this study suggest that most surgeons include their patients’ expectations regarding an active lifestyle in their standard assessment, and, at this time, patients could be encouraged to achieve higher levels of physical activity as observed complications associated with RTS are deemed minimal. High expectations for RTS can influence some of the surgical decision-making, especially with regard to femoral head diameters, bearing surfaces, and implant fixation. With improved implant materials and surgical techniques, we observed recommendations for low-impact sports and increasingly liberal recommendations for high-impact sports after THA. Future studies and updated implant registry data will show whether these developments are justified.

## Electronic supplementary material

Below is the link to the electronic supplementary material.

Supplementary file1 (PDF 337 KB)

Supplementary file2 (PDF 339 KB)

Supplementary file3 (R 81 KB)

## Abbreviations

ALTR: Adverse local tissue reactions
ALVAL: Aseptic lymphocytic vasculitis-associated lesions
BMI: Body mass index
CoC: Ceramic on ceramic
HXLPE: Highly cross-linked polyethylene
MoM: Metal on metal
MoHXLPE: Metal on highly cross-linked polyethylene
OA: Osteoarthritis
PoC: Polyethylene on ceramic
RPA: Return to physical activity
RTS: Return to sports
THA: Total hip arthroplasty
TKA: Total knee arthroplasty
