# Supplementary material for: Recommendations for return to sports after total hip arthroplasty are becoming less restrictive as implants improve
Source: Arch Orthop Trauma Surg. 2020 Dec 1;141(3):497–507. doi: 10.1007/s00402-020-03691-1 (PMC7899958; doi:10.1007/s00402-020-03691-1)
Supplement: Supplementary file 1 — Supplementary file1 (PDF 337 KB) [file 402_2020_3691_MOESM1_ESM.pdf]

DEUTSCHE GESELLSCHAFT FÜR ENDOPROTHETIK

A SURVEY OF THE AE – GERMAN SOCIETY FOR ARTHROPLASTY E.V. TO ASSESS CURRENT RECOMMENDATIONS FOR RETURN TO SPORT AFTER JOINT REPLACEMENT

Please return your completed survey form at the registration or via mail to
Dr. med. PhD Tu-Lan Vu-Han Centrum für Muskuloskeletale Chirurgie (CMSC) | Charité – Universitätsmedizin Berlin, Charitéplatz 1 | 10117 Berlin | Tel. +49 30 450 615 145, E-Mail: tu-lan.vu-han@charite.de
– We thank you for your participation!

O. Voluntary Information

Name:____ Age: ___________

Place of practice:
____

1 General Questions

1.1 "In which year did you receive your Approbation?"
O vor 1980
O 1980 -1990
O 1990 - 2000
O 2000 - 2010
O 2010 – 2020

1.2 "How high do you rate your own athletic activity?"
O "very high, I do sports daily"
O "high, I do sports multiple times a week"O mittel, ich mache ab und zu Sport
O "medium, I do sports every now and then"
O "low, I do not do sports"

1.3 "Is the question of the patient's sporting activity before joint replacement part of your standard patient interview?"
O „Yes“
O “No”
O “I do not know”
O “It does not matter“

1.4 "Which preoperative parameters are relevant for you with regard to postoperative sporting ability in the assessment?"
O „Age“
O „Gender“
O „BMI“
O „Muscle mass“
O "Coordination/Experience of the sport before planned surgery"
O "Bone density"
O "Neurologic precondition "
O "Rheumatologic precondition"

1.5 "What do you think is the **main** risk from sport after implantation of an endoprosthesis? (Maximum 2 answers)"
O "Joint dislocation"
O "Periprosthetic fracture"
O "Implant loosening"
O "Polyethylene-wear"
O "Periprosthetic infection"
O "Osteolysis"
O "Material breach"
O "Tendon rupture"
O „Other _____________________________ (which?)”

2 Specific questions regarding hip arthroplasty

2.1 "How important do you think is athletic activity after hip replacement?"

O "very important"
O “important”
O “not important“
O „does not matter“

2.2 "Do you think that physical activity has a negative impact on the durability of a hip arthroplasty (reduced lifespan)?"

O „Yes“
O „No“
O „I don’t know“
O „does not matter“

2.3 "Do you think that physical activity has a positive impact on the durability of a hip arthroplasty (reduced lifespan)?"

O „Yes“
O „No“
O „I do not know“
O „It does not matter“

2.4 "The burden that patients with a hip arthroplasty expose themselves to is usually ..."

O "Way too high"
O "A little too high"
O "Just right"
O "Too low"
O "Way too low"

2.5 "How often do you attribute failure of hip arthroplasty to stress overload or sports?"

O "In more than 50% of revision surgeries"
O "In more than 25% of revision surgeries"
O "In more than 10% of revision surgeries"
O "In more than 5% of revision surgeries"
O "In more than 1% of revision surgeries"
O "In less than 1% of revision surgeries"

2.6 "Does a high level of sporting ability influence the choice of your surgical approach?"

O “Yes,
if yes, which one would you prefer?“
  O anteriorer surgical approach
  O lateral surgical approach
  O anterolateraler surgical approach
  O dorsaler surgical approach
O No

2.7 "Does the desired sport influence the choice of implant fixation of the hip arthroplasty? If yes, which one do you prefer?"

O cementless
O cemented
O hybrid
O does not matter

2.8 "Would you deviate from your standard positioning of the hip arthroplasty when a sport activity with high range of motion is expected?"

O Yes,
  if yes, which do you prefer?
  O "Yes, increased inclination "
  O "Yes, reduced inclination"
  O "Yes, increased stem anteversion"
  O "Yes, reduced cup anteversion"
  O „other“
O No

2.9 "Which material pairings do you prefer to use depending on the level of sporting activity mentioned below?"

Materialgleitpaarung	High-Impact*	Low-Impact*	No Sport
Metal on Metall (MoM)	o	o	o
Metal on Polyethylen (MoHXLPE)	o	o	o
Ceramic on Polyethylen (CoHXLPE)	o	o	o
Ceramic on Ceramic (CoC)	o	o	o
Egal	o	o	o

2.10 "Which shaft type do you prefer for patients with high sporting activity?"
O „short stem“
O „straight/standard stem”
O “does not matter”

2.11 "Which head size do you prefer to use depending on the sporting activity mentioned below?"

Femoral head size	High-Impact*	Low-Impact*	No Sport
28mm	o	o	o
32mm	o	o	o
36mm	o	o	o
does not matter	o	o	o

2.12 The patient specifies to do sports with extensive range of motion (e.g. yoga) - what criteria do you consider when planning hip replacement? "
O surgical approach_________________________ (welcher?)
O femoral head diameter: ______________________________ (welche?)
O bearing surfaces: _____________________________ (welche?)

2.13 "Your patient expresses the wish to return to previously practiced high-impact sports after the hip arthroplasty surgery. Which statement do you make?"
O "I recommend it without limitations after a certain time of recovery”
O "I recommend it only after adequate training of the patient in regard to movements and risks."
O "I recommend no high-impact sports after arthroplasty"
O "I leave it to the patient and make no specific statement."

2.14 "What is your recommendation for sports after a **total hip arthroplasty (THA)?"**

Type of Sport	"after 3 months"	"after 6 months"	"not recommended"	"undecided"
High-Impact*	O	O	O	O
Low-Impact*	O	O	O	O

[1]

[1] *Low impact sports include swimming, walking, pilates, elliptical training. General characteristics are smooth and even body movements. In contrast, high-impact sports include running, skiing, squash, basketball and boxing. They generally have an increased risk for injury due to abrupt movements.

4. "What is your recommendation for patients after **total hip arthroplasty**?"

Sportart	without limitation	with training	not recommended	undecided
“Basketball”	O	O	O	O
“Bowling”	O	O	O	O
"Boxing/Martial Arts"	O	O	O	O
"E-Scooter"	O	O	O	O
"Fitness/Weights"	O	O	O	O
"Soccer"	O	O	O	O
"Ballroom Dancing"	O	O	O	O
"Golf"	O	O	O	O
"Handball"	O	O	O	O
"Hockey"	O	O	O	O
"Jogging"	O	O	O	O
"Climbing"	O	O	O	O
"Pilates"	O	O	O	O
"Biking level"	O	O	O	O
"Biking cross"	O	O	O	O
"Horseback riding"	O	O	O	O
"Rowing"	O	O	O	O
"Swimming"	O	O	O	O
"Ski slope"	O	O	O	O
"Ski long"	O	O	O	O
"Walking"	O	O	O	O
“Squash”	O	O	O	O
"Surfing"	O	O	O	O
“Dancing"	O	O	O	O
"Tennis"	O	O	O	O
"Table tennis"	O	O	O	O
"Gymnastics"	O	O	O	O
“Volleyball”	O	O	O	O
“Hiking”	O	O	O	O
“Yoga”	O	O	O	O

Please return your questionnaire at the reception or via e-mail to
Dr. med. Tu-Lan Vu-Han, PhD Centrum für Muskuloskeletale Chirurgie (CMSC) | Charité – Universitätsmedizin Berlin, Chariteplatz 1 | 10117 Berlin | Tel. +49 30 450 615 145, E-Mail: tu-lan.vu-han@charite.de
– We thank you for your participation!
