# Supplementary material for: Recommendations for return to sports after total hip arthroplasty are becoming less restrictive as implants improve
Source: Arch Orthop Trauma Surg. 2020 Dec 1;141(3):497–507. doi: 10.1007/s00402-020-03691-1 (PMC7899958; doi:10.1007/s00402-020-03691-1)
Supplement: Supplementary file 2 — Supplementary file2 (PDF 339 KB) [file 402_2020_3691_MOESM2_ESM.pdf]

DEUTSCHE GESELLSCHAFT FÜR ENDOPROTHETIK

EINE UMFRAGE DER AE - DEUTSCHE GESELLSCHAFT FÜR ENDOPROTHETIK E.V. ZUR EINSCHÄTZUNG DER SPORTLICHEN BELASTUNGSFÄHIGKEIT VON PATIENTEN NACH GELENKIMPLANTATIONEN

– Wir danken Ihnen für Ihre geschätzte Teilnahme!

O. Freiwillige Angaben

Name:____ Alter: ___________

Tätigkeitsort: ____

1 Allgemeine Fragen

1.1 In welchem Jahr ca. haben Sie ihre Approbation erhalten?
- O vor 1980
- O 1980 -1990
- O 1990 - 2000
- O 2000 - 2010
- O 2010 – 2020

1.2 Wie hoch schätzen Sie ihre eigene sportliche Aktivität ein?
- O sehr hoch, ich mache täglich Sport
- O hoch, ich mache mehrmals die Woche Sport
- O mittel, ich mache ab und zu Sport
- O niedrig, ich mache gar kein Sport

1.3 Gehört die Frage nach der sportlichen Aktivität des Patienten vor Endoprothesenersatz zu Ihrem Standardfragekatalog?
- O Ja
- O Nein
- O Weiß nicht
- O egal

1.4 Welche **präoperativen Parameter** sind für Sie hinsichtlich der postoperativen Sportfähigkeit in der Beurteilung relevant?
- O Alter
- O Geschlecht
- O BMI
- O Muskelmasse
- O Koordination/Ausübung der Sportart vor der geplanten Operation
- O Knochendichte

O neurologische Nebenerkrankungen
O rheumatologische Grunderkrankungen

1.5 Welches ist Ihrer Einschätzung nach das **Hauptrisiko** durch Sport nach Implantation einer Endoprothese?
(maximal 2 Nennungen)
- O Luxation
- O Periprothetische Fraktur
- O vorzeitige Prothesenlockerung
- O PE-Abrieb
- O Periprothetische Infektion
- O Osteolyse
- O Materialbruch
- O Bandruptur
- O Andere ______________________________ (welche?)

2 Spezielle Fragen zum Gelenkersatz an der Hüfte

2.1 Für wie wichtig halten Sie sportliche Aktivität nach **Hüftendoprothesenersatz**?
- O sehr wichtig
- O wichtig
- O nicht wichtig
- O egal

2.2 Glauben Sie, dass sportliche Aktivität die Haltbarkeit einer **Hüftendoprothese** negativ beeinflusst (Standzeit reduziert)?
- O Ja
- O Nein
- O Weiß nicht
- O egal

2.3 Glauben Sie, dass die sportliche Aktivität die Funktion der **Hüftendoprothese** positiv beeinflusst?
- O Ja
- O Nein
- O Weiß nicht
- O egal

2.4 Die **Belastung**, welcher sich Patienten mit einer **Hüftendoprothese** aussetzen, ist in der Regel
- O Viel zu hoch
- O Etwas zu hoch
- O Genau richtig
- O Zu niedrig
- O Viel zu niedrig

2.5 Wie häufig führen Sie **Fehlschläge** von **Hüftendoprothesen** auf Überlastung bzw. Sport zurück?
- O In mehr als 50% der Revisionen
- O In mehr als 25% der Revisionen
- O In mehr als 10% der Revisionen
- O In mehr als 5% der Revisionen
- O In mehr als 1% der Revisionen
- O In weniger als 1% der Revisionen

2.6 Hat ein hoher sportlicher Anspruch Einfluss auf die Wahl ihres **operativen Zugangsweges**?
- O Ja,
  wenn ja, welchen würden sie in diesem Fall nutzen?
  - O anteriorer Zugang
  - O lateral Zugang
  - O anterolateraler Zugang
  - O dorsaler Zugang
- O Nein

2.7 Hat die gewünschte Sportart Einfluss auf die **Wahl der Verankerung** der Hüftendoprothese? Wenn ja, welche wählen Sie bevorzugt?
- O zementfrei
- O zementiert
- O hybrid
- O egal

2.8 Würden Sie bei einer Sportart mit zu erwartenden hohen Bewegungsumfängen von ihrer **Standardpositionierung der Hüftendoprothese abweichen**?
- O Ja,
  wenn ja, welche?
  - O vermehrte Inklination
  - O verminderte Inklination
  - O vermehrte Schaft-Anteversion
  - O verminderte Pfannen-Anteversion
  - O andere
- O Nein

2.9 Welche **Materialgleitpaarungen** verwenden Sie bevorzugt in Abhängigkeit der unten genannten sportlichen Aktivität?

Materialgleitpaarung	High-Impact*	Low-Impact*	Kein Sport
Metal on Metal (MoM)	o	o	o
Metal on Polyethylene (MoHXLPE)	o	o	o
Ceramic on Polyethylene (CoHXLPE)	o	o	o
Ceramic on Ceramic (CoC)	o	o	o
Egal	o	o	o

2.10 Welchen **Schafttyp** wählen Sie bevorzugt bei Patienten mit hoher sportlicher Aktivität?

O Kurzschaft
O Geradschaft
O Egal

2.11 Welche **Kopfgröße** verwenden Sie bevorzugt in Abhängigkeit der unten genannten sportlichen Aktivität?

Kopfgröße	High-Impact*	Low-Impact*	Kein Sport
28mm	o	o	o
32mm	o	o	o
36mm	o	o	o
Egal	o	o	o

2.12 Der Patient gibt an vor allem Sportarten mit endgradigen Bewegungsumfängen (z. B. Yoga) durchzuführen – **welche Kriterien** berücksichtigen Sie bei der Planung des Hüftgelenkersatzes?

O operativer Zugang__________________________ (welcher?)
O Kopfgröße: ________________________________ (welche?)
O Gleitpaarung: ______________________________ (welche?)

2.13 Ihr Patient äußert den Wunsch nach der **Hüft-TEP OP** zu zuvor ausgeübten **high-impact*** Sportarten zurückzukehren. Welche Aussage treffen Sie?

O Ich empfehle es uneingeschränkt nach einer gewissen Schonzeit
O Ich empfehle es nur bei adäquater Schulung des Patienten über die Bewegungen/Risiken
O Ich empfehle keine high-impact Sportarten nach Implantation einer Gelenkendoprothese
O Ich überlasse es dem Patienten und spreche keine konkrete Empfehlung aus

2.14 Wie lautet Ihre **Sport Empfehlung** nach Implantation einer **Hüft-Totalendoprothese (Hüft-TEP)?**

Sportart	n. 3 Monaten	n. 6 Monaten	Nicht empfohlen	Unentschieden
High-Impact*	O	O	O	O
Low-Impact*	O	O	O	O

[1]

[1] *Zu den Low-Impact Sportarten zählen u.a. Schwimmen, Gehen, Pilates und elliptisches Kardiotraining (Crosstrainer). Sie zeichnen sich durch sanfte und flüssige Bewegungen aus, bei denen das Verletzungsrisiko gering ist. Zu den High-Impact Sportarten zählen u.a. Laufen, Skifahren, Squash, Basketball und Boxen, bei denen das Verletzungsrisiko hoch ist, insbesondere, wenn Patienten nicht trainiert oder ungeschult sind.

4. Wie lautet Ihre Empfehlung für Patienten nach **Hüft-Total-Endoprothesenersatz**?

**Sportart**	uneingeschränkt empfohlen	nur mit Schulung	nicht empfohlen	unentschieden
Basketball	O	O	O	O
Bowling	O	O	O	O
Boxing/Kampfsport	O	O	O	O
E-Scooter fahren	O	O	O	O
Fitness/Gewichte Heben	O	O	O	O
Fußball	O	O	O	O
Gesellschaftstanz (Partner)	O	O	O	O
Golf	O	O	O	O
Handball	O	O	O	O
Hockey	O	O	O	O
Joggen	O	O	O	O
Klettern	O	O	O	O
Pilates	O	O	O	O
Radfahren Ebene	O	O	O	O
Radfahren Unebene	O	O	O	O
Reiten	O	O	O	O
Rudern	O	O	O	O
Schwimmen	O	O	O	O
Skifahren (alpin)	O	O	O	O
Skilanglauf	O	O	O	O
Spazieren	O	O	O	O
Squash	O	O	O	O
Surfen	O	O	O	O
Tanzen	O	O	O	O
Tennis	O	O	O	O
Tischtennis	O	O	O	O
Turnen	O	O	O	O
Volleyball	O	O	O	O
Wandern	O	O	O	O
Yoga	O	O	O	O
